# The Calcium-Induced Regulation in the Molecular and Transcriptional Circuitry of Human Inflammatory Response and Autoimmunity

**DOI:** 10.3389/fphar.2017.00962

**Published:** 2018-01-08

**Authors:** Ivo R. de Seabra Rodrigues Dias, Simon W. F. Mok, Flora Gordillo-Martínez, Imran Khan, Wendy W. L. Hsiao, Betty Y. K. Law, Vincent K. W. Wong, Liang Liu

**Affiliations:** State Key Laboratory of Quality Research in Chinese Medicine, Macau University of Science and Technology, Macau, China

**Keywords:** Rheumatoid arthritis, RASFs, celastrol, calcium, inflammation, autoimmunity

## Abstract

Rheumatoid arthritis synovial fibroblasts (RASFs) are fundamental effector cells in RA driving the joint inflammation and deformities. Celastrol is a natural compound that exhibits a potent anti-arthritic effect promoting endoplasmic reticulum (ER) stress mediated by intracellular calcium (Ca^2+^) mobilization. Ca^2+^ is a second messenger regulating a variety of cellular processes. We hypothesized that the compound, celastrol, affecting cytosolic Ca^2+^ mobilization could serve as a novel strategy to combat RA. To address this issue, celastrol was used as a molecular tool to assay the inflammatory gene expression profile regulated by Ca^2+^. We confirmed that celastrol treatment mobilized cytosolic Ca^2+^ in patient-derived RASFs. It was found that 23 genes out of 370 were manipulated by Ca^2+^ mobilization using an inflammatory and autoimmunity PCR array following independent quantitative PCR validation. Most of the identified genes were downregulated and categorized into five groups corresponding to their cellular responses participating in RA pathogenesis. Accordingly, a signaling network map demonstrating the possible molecular circuitry connecting the functions of the products of these genes was generated based on literature review. In addition, a bioinformatics analysis revealed that celastrol-induced Ca^2+^ mobilization gene expression profile showed a novel mode of action compared with three FDA-approved rheumatic drugs (methotrexate, rituximab and tocilizumab). To the best of our knowledge, this is a pioneer work charting the Ca^2+^ signaling network on the regulation of RA-associated inflammatory gene expression.

## Introduction

Rheumatoid arthritis (RA) is the most common chronic systemic autoimmune inflammatory disease. RA affects around 1% of people worldwide, and is more prevalent in women (Bartok and Firestein, 2010; Burmester and Pope, 2017). The quality of life in RA patients is critically compromised, which usually causes progressive articular destruction, early unemployment, and considerable disability implying a huge socioeconomic burden (Albers et al., 1999; Russell, 2008). RA is characterized by synovitis associated with the formation of a hyperplastic synovial membrane, which contains large number of immunocellular components including T and B cells, plasma cells, mast cells, macrophages, and activated RA synovial fibroblasts (RASFs) (Bartok and Firestein, 2010). The cellular interplay between these cells transforms the synovium into an invasive pannus and promotes angiogenesis, as well as the release of cytokines, chemokines, and matrix-degrading enzymes which facilitates the development of joint tissue damage. In particular, the RASFs, which actively proliferate and develop resistance to apoptosis are the major cell population in the synovial lesion (Perlman et al., 2001; Firestein, 2003; Muller-Ladner et al., 2007; Bartok and Firestein, 2010). These factors, coupled with activated RASFs' capability of secreting the aforementioned mediators of RA, establishes a paracrine/autocrine cycle that perpetuates RA synovitis, recruitment of new cells to the affected joint and induces joint destruction (Bartok and Firestein, 2010), which represents the potential therapeutic target of RASFs for achieving long-term remission of RA.

Early diagnosis and treatment is critical to the diminution of the seriousness of RA. Currently, non-steroidal anti-inflammatory drugs (NSAIDs), glucocorticoids (steroid hormones), and disease-modifying antirheumatic drugs (DMARDs) are standard pharmaceutical interventions for the inflammatory disorder. Of note, DMARDs medications suppress immune system functions to prevent further structural damage of bone and cartilage in the affected joints (Burmester and Pope, 2017; McInnes and Schett, 2017). Amongst which methotrexate (MTX) is the first-line therapy commonly prescribed, and the addition of targeted DMARD, such as tumor necrosis factor (TNF)-inhibitors, interleukin-6-inhibitors, B-cell depleting drugs, and Janus kinase (JAK)-inhibitors, are adjuvant interventions when poor prognostic factors are present. These new treat-to-target medications have improved the course of RA and most of them modulate specific mechanistic steps in the inflammatory process. However, a considerable numbers of individuals do not respond adequately to or are suffering from side-effects toward such treatments (Burmester and Pope, 2017; Tarp et al., 2017). Therefore, a better understanding of the molecular machinery associated with RA pathogenesis and the complex crosstalk between the constituting signaling pathways might help to facilitate the development or improvement of novel and conventional therapeutic strategies.

The Chinese medicinal herb *Tripterygium wilfordii Hook f* (TwHF) is a traditional remedy for RA treatment (Tao et al., 2002; Tang and Zuo, 2012). In fact, the recent randomized clinical trial studies further confirmed the therapeutic efficacy of TwHF in patients with active RA (Lv et al., 2015). Celastrol is the bioactive ingredient constituting TwHF which has demonstrated anti-proliferative and anti-inflammatory properties in both *in vitro* and *in vivo* models (Brinker et al., 2007; Kim et al., 2009; Venkatesha et al., 2011; Cascão et al., 2012; Nanjundaiah et al., 2012). It has been shown that celastrol promotes endoplasmic reticulum (ER) stress mediated by intracellular calcium (Ca^2+^) mobilization (Yoon et al., 2014). Ca^2+^ as a second messenger is required for the regulation of many cellular processes, including gene transcription, cell shape, motility, proliferation, mitochondrial function, apoptosis, and immune responses (Clapham, 2007). As early as two decades ago, the role of cellular Ca^2+^ in diseases was being noticed, from cardiovascular diseases to strokes, diabetes, the immune response (including inflammation) and cancer (Mooren and Kinne, 1998). More recently, studies have shown that intracellular Ca^2+^ signaling has been implicated in the pathogenesis of autoimmune disorders, such as RA (Izquierdo et al., 2014) and that altered cellular Ca^2+^ homeostasis is related to the control of various hallmarks of cancer. (Marchi and Pinton, 2016). Modulation of Ca^2+^ signaling has even been proposed as a possible therapy option for the treatment of cancer, though this remains as yet relatively unexplored (Rooke, 2014). Accordingly, celastrol is a suitable tool for investigating the role of Ca^2+^ signaling in pathomechanisms of RA. In this report, we hypothesized that compounds affecting cytosolic Ca^2+^ mobilization might serve to combat RA disease. The inflammatory gene expression profile which are regulated by Ca^2+^ dynamic changes in response to celastrol treatment were identified in patient-derived RASF. In addition, the possible transcriptional and molecular circuitry associated with the cytosolic Ca^2+^ mobilization was unraveled through literature review and bioinformatics analysis. Our findings provide novel insight into the application of ion channel modulators for RA intervention.

## Materials and methods

### Isolation and culture of RASF

Primary culture RASF were isolated from synovium obtained from RA patients who had undergone knee surgery for synovectomy. Diagnosis of RA in these patients was made according to the American Rheumatism Association's 1987 revised criteria for classification of RA (Arnett et al., 1988). Synovial strips were cut into small pieces, placed in a 25 cm^2^ culture flask, and then cultured in DMEM containing 20% fetal bovine serum (FBS). Medium was changed every 3 days and, after 2 weeks, the synovial tissues were removed from the cultured medium. RASFs were digested by 0.25% trypsin for 5 min at 37°C. The cell suspension was diluted with DMEM containing 20% FBS and 1% Penicillin/Streptomycin with L-Glutamine (PSG), and separated into other flasks. Cell cultures were maintained at 37°C in a humidified incubator (atmosphere of 5% CO_2_). The purification of RASFs was validated by staining CD90. Cultured RASFs from passages 5–7 were employed for the below analysis. Unless otherwise specified, all reagents were purchased from Sigma-Aldrich (MO, USA).

### Measurement of cytoplasmic calcium dynamic

Intracellular cytosolic Ca^2+^ dynamic was measured using the FLIPR Calcium 6 Assay Kit (Molecular Devices, USA), which contains a proprietary Ca^2+^-sensitive fluorophore, according to the manufacturer's instructions. In brief, 10000 RASFs per well were seeded in black wall/clear bottom 96-multiwell plates from Costar (Tewksbury, MA, USA) and cultured for 24 h before treatment. After that, calcium 6 reagent was added directly to cells, and cells were incubated for an additional 2 h at 37°C and 5% CO_2_. One micromolar of celastrol (China Chengdu MUST, A000106) was then added to the wells and immediately subjected to data acquisition on the FLIPR Tetra High-Throughput Cellular Screening System (Molecular Devices, USA) at room temperature using a 1-s reading interval throughout the experiments.

### Single cell calcium imaging

2 × 10^5^ RASFs cells were cultured in 35 mm confocal disc at 37°C CO_2_ incubator for 24 h. FLIPR Calcium 6 reagent was added to cells at 37°C for 30 min. RASFs were then washed 3 times with HEPES buffer saline and incubated at 37°C in an imaging chamber for another 10 min. Changes in cytosolic [Ca^2+^] levels were monitored by following changes in FLIPR Calcium 6 fluorescence upon addition of 1 μM celastrol in HBSS buffer, using the real-time mode for 4 min by epifluorescence microscopy (Applied Precision DeltaVision Elite, Applied Precision, Inc., USA). Data Inspection Program provided by the DeltaVision software was used to measure the intensity of the FLIPR Calcium 6 fluorescence and the mean fluorescence intensity was monitored at 525 nm and plotted against time (s).

### RNA extraction & cDNA synthesis

RNA was extracted using RNeasy Mini Kit (Qiagen, USA) from RASFs untreated (control), treated with celastrol [1 μM], or celastrol in the presence of BAPTA/AM [10 μM] (Santa Cruz, USA) for 24 h. RNA concentration was determined using the NanoDrop 2000c Spectrophotometer (Thermo Scientific) and 1 μg of RNA was used to synthesize cDNA with RT2 First Strand Kit (Qiagen, USA). Three independent biological samples were employed.

### RT^2^ profiler™ PCR array–inflammatory gene expression profiling

Quantitative PCR was performed using the cDNA prepared from RASFs with RT2 SYBR® Green qPCR Mastermix (Qiagen, USA) and ViiA™ 7 Real Time PCR System (Applied Biosystems). The RT^2^ Profiler™ PCR Array Human Inflammatory Response & Autoimmunity 384HT kit (Qiagen, USA) was assessed according to the manufacturer's instructions. This PCR array contains primers for 370 genes related to various functions of the immune system, from cytokines/chemokines to their receptors, cytokine production, and other proteins and receptors involved in acute-phase, inflammatory and humoral immune responses. Analysis of results was achieved using the integrated web-based RT^2^ Profiler™ PCR Array Data Analysis software from Qiagen, which calculated all ΔΔCt-based fold-change and fold-regulation from three independent raw data. Validation of the identified gene expression pattern upon celastrol-induced calcium mobilization was performed by quantitative PCR with self-designed primers (Tech Dragon Ltd., Hong Kong). Primer sequences (see Supplementary Table) were designed employing ThermoFisher Scientific's online OligoPerfect™ Designer software and then verified with NCBI's Primer-BLAST software to confirm specific recognition of target genes. Gene expressions were normalized to GAPDH, relative to control, and analyzed using the 2^−ΔΔCT^ method. All the data were statistically analyzed by unpaired *t*-test.

### Bioinformatics analysis

The identified celastrol-mediated Ca^2+^ mobilized genes in this report were searched for their implication in Ca^2+^-dependent function using NCBI database and KEGG pathways. Furthermore, NCBI database (Geo DataSets) was searched for datasets that were generated by treating RA patient with FDA-approved drugs. Three studies were selected that evaluated Rituximab (GDS4903), Tocilizumab (GDS5068), and Methotrexate (GDS5069) for RA treatment. The gene expression profile was transformed into fold change (2^−ΔΔCT^ method) and merged with our dataset (celastrol-mediated Ca^2+^ mobilization regulated genes) using R (3.3.2). Merged datasets was then processed with Phyloseq (1.19.1) and ggplot2 (2.2.1) packages to generate principal coordinate plot with weighted UniFrac distance. Additionally, SIMPER analysis was performed with R (3.3.2) using vegan (2.4-3) to determine genes that are similarly responding to celastrol [Ca^2+^] and FDA-approved drugs (Rituximab, Tocilizumab, and Methotrexate).

## Results

### Celastrol modulated inflammatory and immunity genes via Ca^2+^ mobilization

**Figure 1:**
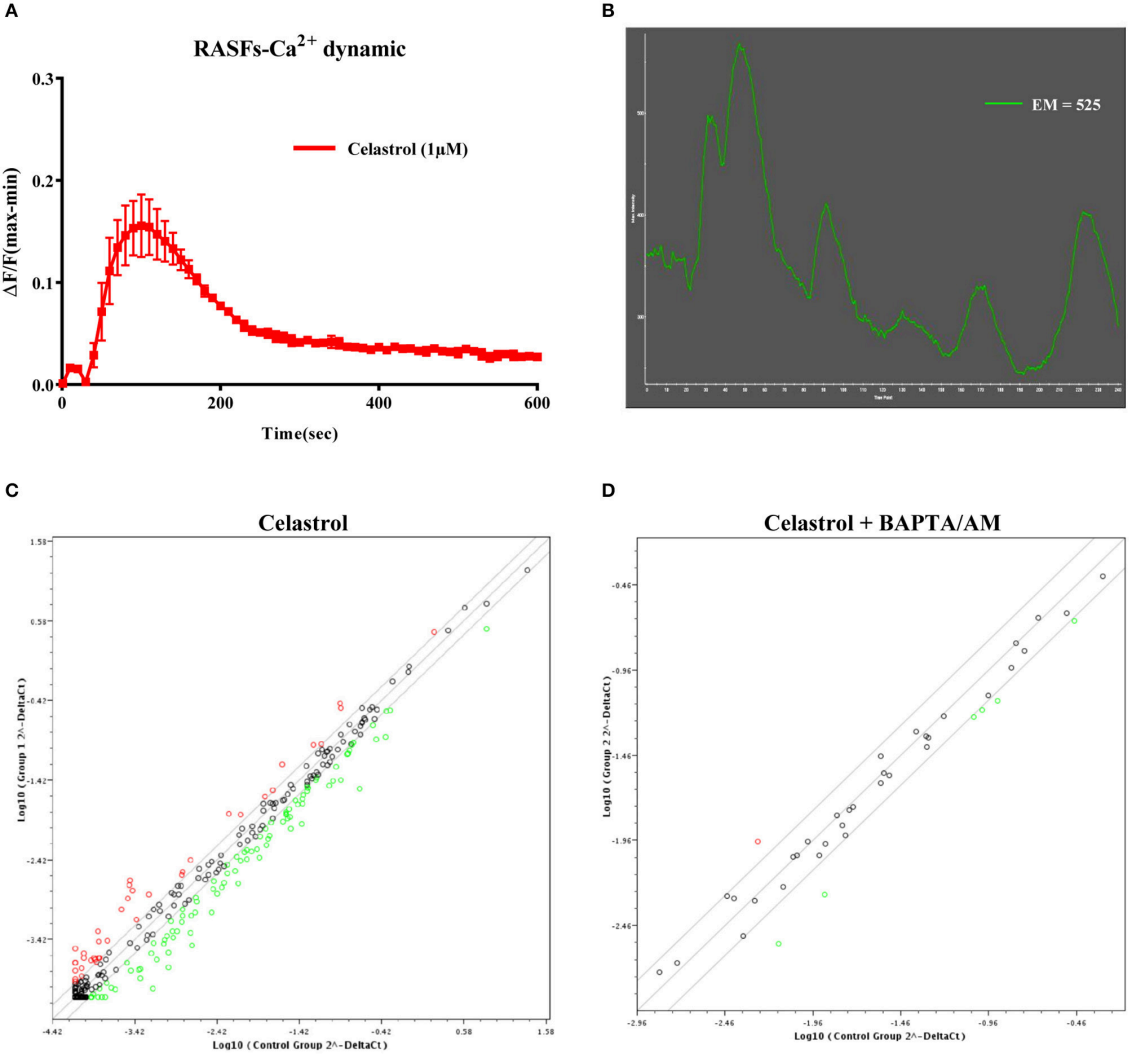
**(A)** Celastrol induced calcium dynamic change in RASFs. Cells treated with 1 μM celastrol were loaded with FLIPR Calcium 6 dye. Real time Ca^2+^ kinetic was monitored with FLIPR Tetra instrument. Data from the chart represent mean values ± SD. of three independent experiments. **(B)** Single cell imaging visualized celastrol-mobilized cytosolic calcium level in RASFs. Cells treated with 1 μM celastrol were loaded with FLIPR Calcium 6 dye. Calcium signal was monitored by Applied Precision DeltaVision Elite in real-time mode (see Supplementary Video). Chart represents the mean intensity of fluorescence signal at 525 nm. **(C)** Scatter plot for inflammatory and immunity genes fold regulation values from celastrol stimulated RASFs relative to unstimulated RASFs (Control): genes not regulated (black), up-regulated genes (red), and down-regulated genes (green) with threshold lines of 1.5 and −1.5. **(D)** Scatter plot for the genes identified as up-regulated or down-regulated with celastrol treatment (in C). Dots represent the genes fold regulation values from RASFs treated with celastrol and BAPTA/AM relative to untreated control: genes not regulated (black), up-regulated genes (red), and down-regulated genes (green) with threshold lines of 1.5 and −1.5 (Ct < 30). Data from the scatter plots represent mean values of three independent experiments.

**Figure 2:**
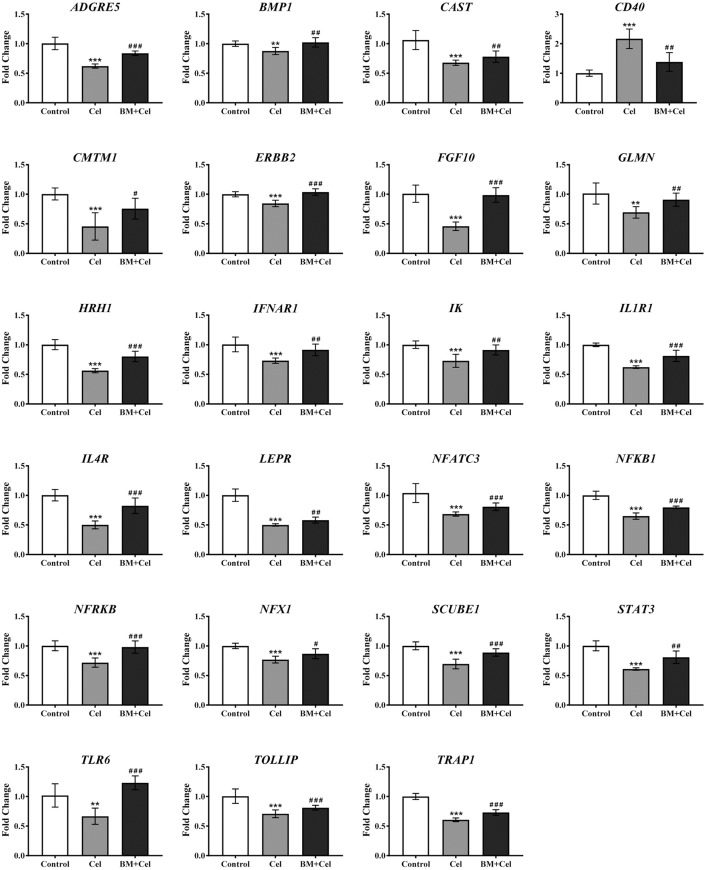
Gene expression regulated by celastrol-mediated Ca^2+^ mobilization analysis in RASF. RT-qPCR independent validation from RASF cells untreated (control), or treated with 1 μM Celastrol (Cel), and 10 μM BAPTA/AM (BM) for 24 h. Gene expressions were normalized to GAPDH, relative to control, and analyzed using the 2^−ΔΔCT^ method. The data is represented as the mean ± SD. ^**^P ≤ 0.01; ^***^P ≤ 0.001 compared with control. ^#^*P* ≤ 0.05; ^##^*P* ≤ 0.01; ^###^*P* ≤ 0.001 compared with Celastrol.

To confirm the effect of celastrol on Ca^2+^ mobilization in RASFs, intracellular cytosolic Ca^2+^ dynamic and single live-cell Ca^2+^ imaging was performed on celastrol-stimulated RASFs. As shown in Figures 1A,B and Supplementary Video, RASFs loaded with FLIPR Calcium 6 displayed a dramatic increase in fluorescence intensity upon 1 μM of celastrol treatment. Celastrol induced Ca^2+^ dynamic changes in RASFs within c.a. 30 s, confirming that celastrol is a suitable tool for Ca^2+^-flux effects studies. Quantitative PCR was performed to identify the inflammatory genes expression profile affected by celastrol-mediated Ca^2+^ mobilization with the use of RT2 Profiler™ PCR Array Human Inflammatory Response & Autoimmunity. Only those genes with changes in fold regulation above ±1.5-fold in the PCR array result were considered to analyse in this study. After celastrol treatment, the expression of 134 out of the total 370 examined genes were up-regulated or down-regulated Figure 1C. Since, the exact mechanistic regulations that mediate such genes expression are still elusive, we investigated the potential role of Ca^2+^ in the expression of these genes. In the presence of Ca^2+^ chelator, BAPTA/AM, we identified that 72 out of the 134 genes were regulated by celastrol-mediated Ca^2+^ mobilization. In addition, those genes which commenced their expression at Ct ≥ 30 were excluded decreasing the number of validated genes to 40 Figure 1D. In order to confirm the reliability of the PCR array result, these 40 genes were further individually validated by quantitative PCR using in-house designed primers. We found that the expression profiles (measured in fold change instead of fold regulation) of 23 out of the 40 genes are consistent with the data observed in the PCR array (Table 1). Of note, celastrol downregulated almost all of the genes (22 of them) under examination in a cytosolic Ca^2+^-dependent manner except CD40 which was upregulated in response to the changes in cellular Ca^2+^ level (Figure 2).

**Table 1:** Gene expression (fold change relative to untreated control) regulated by celastrol-mediated Ca^2+^ mobilization analysis in RASFs.

**Gene ID**	**QiagenRT**^**2**^ **PCR Array**		**RT-qPCR**	
	**Celastrol**	**Celastrol + BAPTA/AM**	**Celastrol**	**Celastrol + BAPTA/AM**
*ADGRE5*	0.3176	0.5898	0.6211 (*P* < 0.0001)	0.8387 (*P* < 0.0001)
*BMP1*	0.3775	0.6336	0.8753 (*P* = 0.0025)	1.0220 (*P* = 0.0050)
*CAST*	0.6147	0.7776	0.6776 (*P* < 0.0001)	0.7807 (*P* = 0.0031)
*CD40*	2.7572	2.0191	2.1670 (*P* < 0.0001)	1.3830 (*P* = 0.0018)
*CMTM1*	0.3232	0.4100	0.4559 (*P* = 0.0003)	0.7562 (*P* = 0.0302)
*ERBB2*	0.4435	0.8811	0.8453 (*P* = 0.0003)	1.0370 (*P* = 0.0001)
*FGF10*	0.3967	0.6883	0.4591 (*P* < 0.0001)	0.9871 (*P* < 0.0001)
*GLMN*	0.5944	0.8093	0.6939 (*P* = 0.0032)	0.9082 (*P* = 0.0050)
*HRH1*	0.4621	0.9831	0.5625 (*P* < 0.0001)	0.8033 (*P* < 0.0001)
*IFNAR1*	0.6417	0.9660	0.7304 (*P* = 0.0004)	0.9142 (*P* = 0.0019)
*IK*	0.6506	0.8039	0.7291 (*P* = 0.0004)	0.9129 (*P* = 0.0088)
*IL1R1*	0.5524	0.7671	0.6225 (*P* < 0.0001)	0.8133 (*P* = 0.0007)
*IL4R*	0.5308	1.2767	0.5002 (*P* < 0.0001)	0.8250 (*P* = 0.0003)
*LEPR*	0.5051	0.7810	0.4999 (*P* < 0.0001)	0.5823 (*P* = 0.0041)
*NFATC3*	0.5724	0.7983	0.6844 (*P* < 0.0001)	0.8080 (*P* = 0.0002)
*NFKB1*	0.6617	0.9204	0.6493 (*P* < 0.0001)	0.7975 (*P* < 0.0001)
*NFRKB*	0.5786	0.9996	0.7185 (*P* = 0.0001)	0.9821 (*P* = 0.0006)
*NFX1*	0.6264	0.8874	0.7698 (*P* < 0.0001)	0.8681 (*P* = 0.0386)
*SCUBE1*	0.2827	0.3908	0.6950 (*P* < 0.0001)	0.8897 (*P* = 0.0010)
*STAT3*	0.5898	1.0051	0.6101 (*P* < 0.0001)	0.8094 (*P* = 0.0011)
*TLR6*	0.5915	1.4554	0.6660 (*P* = 0.0050)	1.2320 (*P* < 0.0001)
*TOLLIP*	0.5269	1.1267	0.7076 (*P* = 0.0004)	0.8102 (*P* = 0.0086)
*TRAP1*	0.5209	0.7086	0.6071 (*P* < 0.0001)	0.7303 (*P* = 0.0003)

*P values in the Celastrol and Celastrol + BAPTA/AM groups are relative to untreated control and Celastrol-treated groups, respectively*.

### Ca^2+^ modulated genes expression in RASFs were categorized into five RA pathogenic factors

To illustrate the functional roles of the identified Ca^2+^-modulated genes, an intensive literature review was performed. These genes were further categorized into five groups according to their cellular functions as: “Apoptosis/cell death,” “Cell proliferation,” “Cell migration/invasion,” “Angiogenesis,” and “Immunity/inflammation” for analysis (Table 2). Such cellular processes are the key factors etiologically associated with the progress of RA inflammation. The aberrant proliferation (Firestein, 2003) and hampered apoptotic machinery (Perlman et al., 2001) of RASFs are responsible for pannus formation. The formation and progressive invasion of pannus, which consist of mainly RASFs, in RA joints are responsible for the bone and cartilage destruction and supported by extensive vascular overgrowth (Bartok and Firestein, 2010). Also, the spread of the symptoms from the affected site to different joints is related to the abnormal migratory ability of RASFs (Lefèvre et al., 2009). The release of different cytokines and chemokines are critical to attract the circulatory immunocellular components infiltrating the RA-affected synovium (Bartok and Firestein, 2010). The 23 identified genes appeared to be multi-functional and manipulate a multitude of physiological responses of RASFs. For example, the regulatory effects of bone morphogenetic protein 1 (*BMP1*), calpastatin (*CAST*), and Toll-like receptor 6 (*TLR6*), participate in all of the above described cellular functions. Among these genes, 17 of them play a role in apoptosis/cell death, 17 genes regulate cell migration/invasion, 15 genes are involved in angiogenesis, 22 genes can promote or inhibit cell proliferation, and 21 genes are associated with immunity/inflammation. The products of these genes represent a great variety ranging from upstream cellular receptors, e.g., TLR6 and leptin receptor (*LEPR*), signaling molecules like cytokines and enzymes, e.g., IK cytokines (*IK*) and BMP1, to downstream transcriptional factors, e.g., nuclear factor of activated T-cells c3 (*NFATC3*) and signal transducer and activator of transcription 3 (*STAT3*). In addition, apart from the genes *CAST, CD40, NFATC3*, and *TRAP1*, which had previously been found to be regulated by Ca^2+^-signaling machinery (Casanova et al., 2006; Hanna et al., 2008; Landriscina et al., 2010; Brun and Godbout, 2016), the remaining 19 genes were newly discovered to have their expression regulated by cytosolic Ca^2+^.

**Table 2:** Association of the validated calcium-modulated genes with five important RA pathogenesis factors. Genes were categorized by pathogenic factors (enhancement or inhibition) through the use of NCBI database.

**No**.	**Gene**	**Encoded protein**
**APOPTOSIS/CELL DEATH**		
1	*BMP1*	Bone morphogenetic protein 1
2	*CAST*	Calpastatin
3	*CD40*	CD40 molecule
4	*CMTM1*	CKLF like MARVEL transmembrane domain containing 1
5	*ERBB2*	Erb-b2 receptor tyrosine kinase 2
6	*FGF10*	Fibroblast growth factor 10
7	*IFNAR1*	Interferon α and β receptor subunit 1
8	*IL1R1*	Interleukin 1 receptor type 1
9	*IL4R*	Interleukin 4 receptor
10	*LEPR*	Leptin receptor
11	*NFATC3*	Nuclear factor of activated T-cells 3
12	*NFKB1*	Nuclear factor κ B subunit 1
13	*NFRKB*	Nuclear factor related to κB binding protein
14	*STAT3*	Signal transducer and activator of transcription 3
15	*TLR6*	Toll-like receptor 6
16	*TOLLIP*	Toll interacting protein
17	*TRAP1*	TNF receptor associated protein 1
**CELL MIGRATION/INVASION**		
1	*ADGRE5*	Adhesion G protein-coupled receptor E5
2	*BMP1*	Bone morphogenetic protein 1
3	*CAST*	Calpastatin
4	*CD40*	CD40 molecule
5	*CMTM1*	CKLF like MARVEL transmembrane domain containing 1
6	*ERBB2*	Erb-b2 receptor tyrosine kinase 2
7	*FGF10*	Fibroblast growth factor 10
8	*HRH1*	Histamine receptor H1
9	*IL4R*	Interleukin 4 receptor
10	*LEPR*	Leptin receptor
11	*NFATC3*	Nuclear factor of activated T-cells 3
12	*NFKB1*	Nuclear factor κ B subunit 1
13	*NFRKB*	Nuclear factor related to κB binding protein
14	*STAT3*	Signal transducer and activator of transcription 3
15	*TLR6*	Toll-like receptor 6
16	*TOLLIP*	Toll interacting protein
17	*TRAP1*	TNF receptor associated protein 1
**ANGIOGENESIS**		
1	*ADGRE5*	Adhesion G protein-coupled receptor E5
2	*BMP1*	Bone morphogenetic protein 1
3	*CAST*	Calpastatin
4	*ERBB2*	Erb-b2 receptor tyrosine kinase 2
5	*FGF10*	Fibroblast growth factor 10
6	*HRH1*	Histamine receptor H1
7	*IL1R1*	Interleukin 1 receptor type 1
8	*LEPR*	Leptin receptor
9	*NFATC3*	Nuclear factor of activated T-cells 3
10	*NFKB1*	Nuclear factor κ B subunit 1
11	*NFRKB*	Nuclear factor related to κB binding protein
12	*SCUBE1*	Signal peptide, CUB domain, EGF-like domain-containing protein 1
13	*STAT3*	Signal transducer and activator of transcription 3
14	*TLR6*	Toll-like receptor 6
15	*TOLLIP*	Toll interacting protein
**CELL PROLIFERATION**		
1	*ADGRE5*	Adhesion G protein-coupled receptor E5
2	*BMP1*	Bone morphogenetic protein 1
3	*CAST*	Calpastatin
4	*CD40*	CD40 molecule
5	*CMTM1*	CKLF like MARVEL transmembrane domain containing 1
6	*ERBB2*	Erb-b2 receptor tyrosine kinase 2
7	*FGF10*	Fibroblast growth factor 10
8	*GLMN*	Glomulin, FKBP associated protein
9	*HRH1*	Histamine receptor H1
10	*IFNAR1*	Interferon α and β receptor subunit 1
11	*IK*	IK cytokine, down-regulator of HLA II
12	*IL1R1*	Interleukin 1 receptor type 1
13	*IL4R*	Interleukin 4 receptor
14	*LEPR*	Leptin receptor
15	*NFATC3*	Nuclear factor of activated T-cells 3
16	*NFKB1*	Nuclear factor κ B subunit 1
17	*NFRKB*	Nuclear factor related to κB binding protein
18	*NFX1*	Nuclear transcription factor, X-box binding 1
19	*STAT3*	Signal transducer and activator of transcription 3
20	*TLR6*	Toll-like receptor 6
21	*TOLLIP*	Toll interacting protein
22	*TRAP1*	TNF receptor associated protein 1
**IMMUNITY/INFLAMMATION**		
1	*ADGRE5*	Adhesion G protein-coupled receptor E5
2	*BMP1*	Bone morphogenetic protein 1
3	*CAST*	Calpastatin
4	*CD40*	CD40 molecule
5	*ERBB2*	Erb-b2 receptor tyrosine kinase 2
6	*FGF10*	Fibroblast growth factor 10
7	*GLMN*	Glomulin, FKBP associated protein
8	*HRH1*	Histamine receptor H1
9	*IFNAR1*	Interferon α and β receptor subunit 1
10	*IK*	IK cytokine, down-regulator of HLA II
11	*IL1R1*	Interleukin 1 receptor type 1
12	*IL4R*	Interleukin 4 receptor
13	*LEPR*	Leptin receptor
14	*NFATC3*	Nuclear factor of activated T-cells 3
15	*NFKB1*	Nuclear factor κ B subunit 1
16	*NFRKB*	Nuclear factor related to κB binding protein
17	*NFX1*	Nuclear transcription factor, X-box binding 1
18	*STAT3*	Signal transducer and activator of transcription 3
19	*TLR6*	Toll-like receptor 6
20	*TOLLIP*	Toll interacting protein
21	*TRAP1*	TNF receptor associated protein 1

### Perspective signaling network connecting the Ca^2+^-modulated inflammatory genes in RA

**Figure 3:**
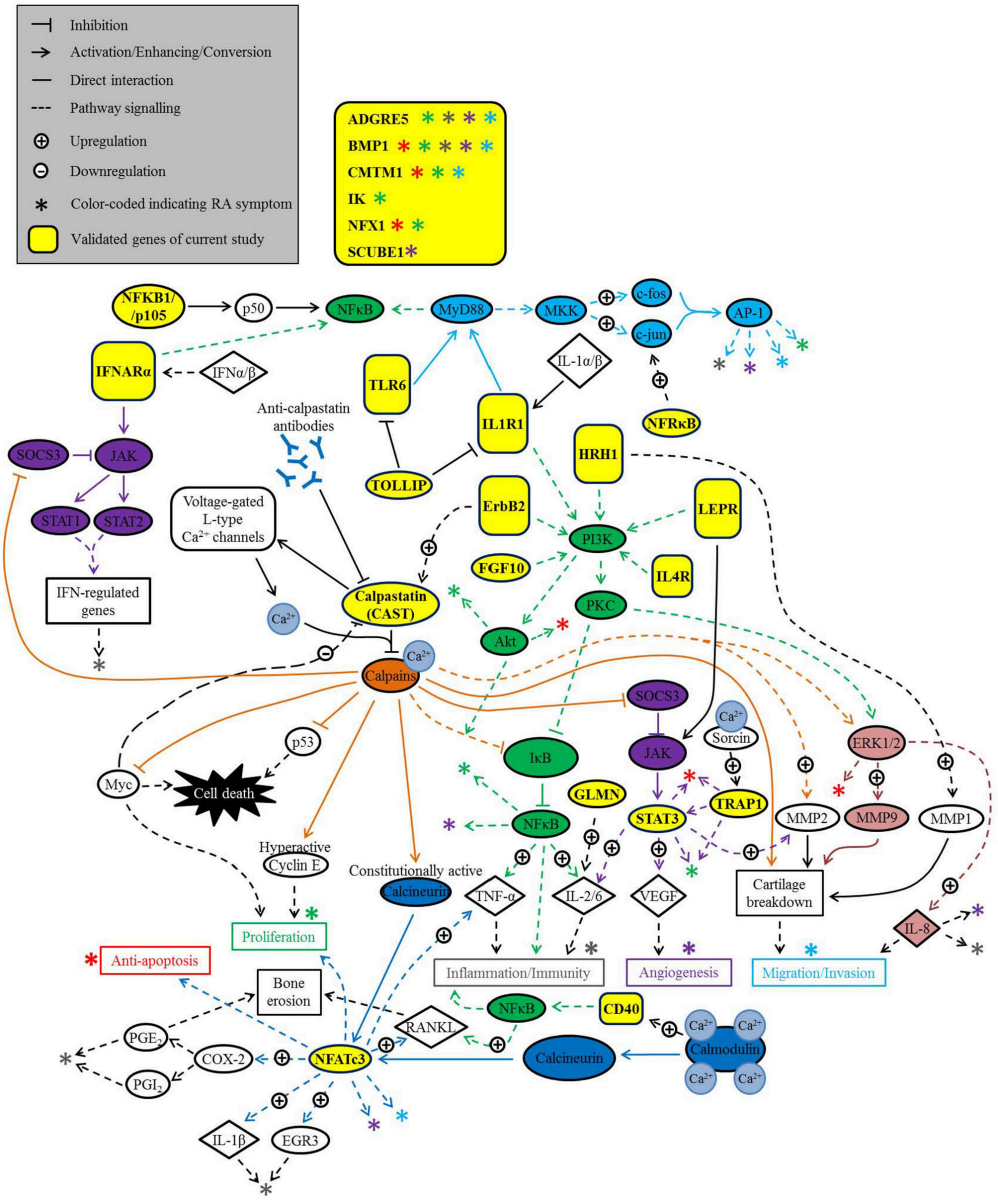
Network map linking some of the Ca^2+^-modulated genes to Ca^2+^signaling pathways and five RA pathogenic factors. Literature review showing only the enhanced effects that drive RA pathogenesis.

Accordingly, a network scheme (Figure 3) involving the identified genes (yellow boxes), as well as the other related genes and pathways were mapped to demonstrate the Ca^2+^ involvement in RA pathogenesis. The network map illustrated major Ca^2+^-signaling pathways: (1) calpastatin (CAST)/calpains (Minobe et al., 2006; Hanna et al., 2008) and (2) calpains/calmodulin-calcineurin-NFATc3 axes (Hernández et al., 2001; Dai et al., 2005; Lee et al., 2011; Li et al., 2011; Neria et al., 2013; Yoon et al., 2013; Jia et al., 2014; Baron et al., 2015; Kar and Parekh, 2015; Brun and Godbout, 2016; Mognol et al., 2016) (blue boxes and arrows). Some pathways that are positively regulated by calpains (orange arrows) are well-known pathways such as NF-κB signaling axis (Li et al., 2011, 2014; Storr et al., 2011, 2015) (green boxes and arrows; includes *NFKB1*, which encodes the precursor protein of p50, one of the subunits of NF-κB, Karin and Ben-Neriah, 2000), JAK/STAT axis (purple boxes and arrows; includes *STAT3*) (Pothlichet et al., 2008; Miyazaki et al., 2015), and the ERK1/2 axis (Moshal et al., 2006) (pale red boxes and arrows). Calpain has been demonstrated to inhibit two apoptotic pathways by cleaving p53 (Storr et al., 2015) and Myc (Niapour et al., 2008; Storr et al., 2011). When released to the extracellular medium, it also promotes proliferation by converting Cyclin E into a hyperactive form (Storr et al., 2011) which may directly break down cartilage (Ishikawa et al., 1999). Another protein, TRAP1, which is stabilized by the Ca^2+^-dependent protein Sorcin, promotes cell proliferation and inhibits apoptosis (Landriscina et al., 2010). The other identified calcium-modulated genes have also been found to activate pathways such as: JAK/STAT axis [*IFNAR1* (Walters and Jelinek, 2004; Qian et al., 2011), *LEPR* (Sanchez-Margalet and Martin-Romero, 2001), and *TRAP1* (Ou et al., 2014)]; PI3K/Akt axis [*ERBB2* (Woods Ignatoski et al., 2003), *IL1R1* (Sizemore et al., 1999), *IL4R* (Dubois et al., 1998), *LEPR* (Uddin et al., 2010), and *FGF10* (Li et al., 2016)] and PI3K/PKC/NF-κB axis [green boxes and arrows; *HRH1*, (Dickenson, 2002) and *ERBB2* (Woods Ignatoski et al., 2003)]; and also NF-κB signaling pathway through other signaling cascades [*CD40* (Lee et al., 2006), *IFNAR1* (Yang et al., 2008), *IL1R1* (Dower et al., 1986; Burns et al., 1998; Ahmad et al., 2007), and *TLR6* (de Almeida et al., 2013)]; and MyD88-dependent MAPKs/AP-1 axis [light blue boxes and arrows; activated by *IL1R1* (Dower et al., 1986; Burns et al., 1998; Ahmad et al., 2007), *TLR6* (de Almeida et al., 2013), and *NFRKB* (Audard et al., 2012)]. The identified gene *TOLLIP* inhibits *IL1R1* and *TLR6* function (Burns et al., 2000; Bulut et al., 2001), and thereby prevents the activation of MyD88-dependent MAPK/AP-1 and NF-κB pathways, as well as PI3K pathways. *GLMN*, encoding the protein glomulin, also known as FAP48 or FAP68, has been shown to upregulate IL-2 expression (Krummrei et al., 2003). Other effects caused by activation of these mentioned genes include *ERBB2* upregulating *CAST* expression (Ai et al., 2013) and *HRH1* upregulating MMP1, possibly through AP-1 activation (Zenmyo et al., 1995). The other identified genes including *ADGRE5, BMP1, CMTM1, IK, NFX1*, and *SCUBE1* are still elusive in an inflammatory pathways. In summary, the network presented in Figure 3 revealed the possible connection between the Ca^2+^-signaling axis and the identified calcium-modulated genes.

### Mode of action of celastrol-induced Ca^2+^-mobilization (celastrol-[Ca^2+^]) was unique in modulating gene expression compared with the FDA-approved RA drugs

**Figure 4:**
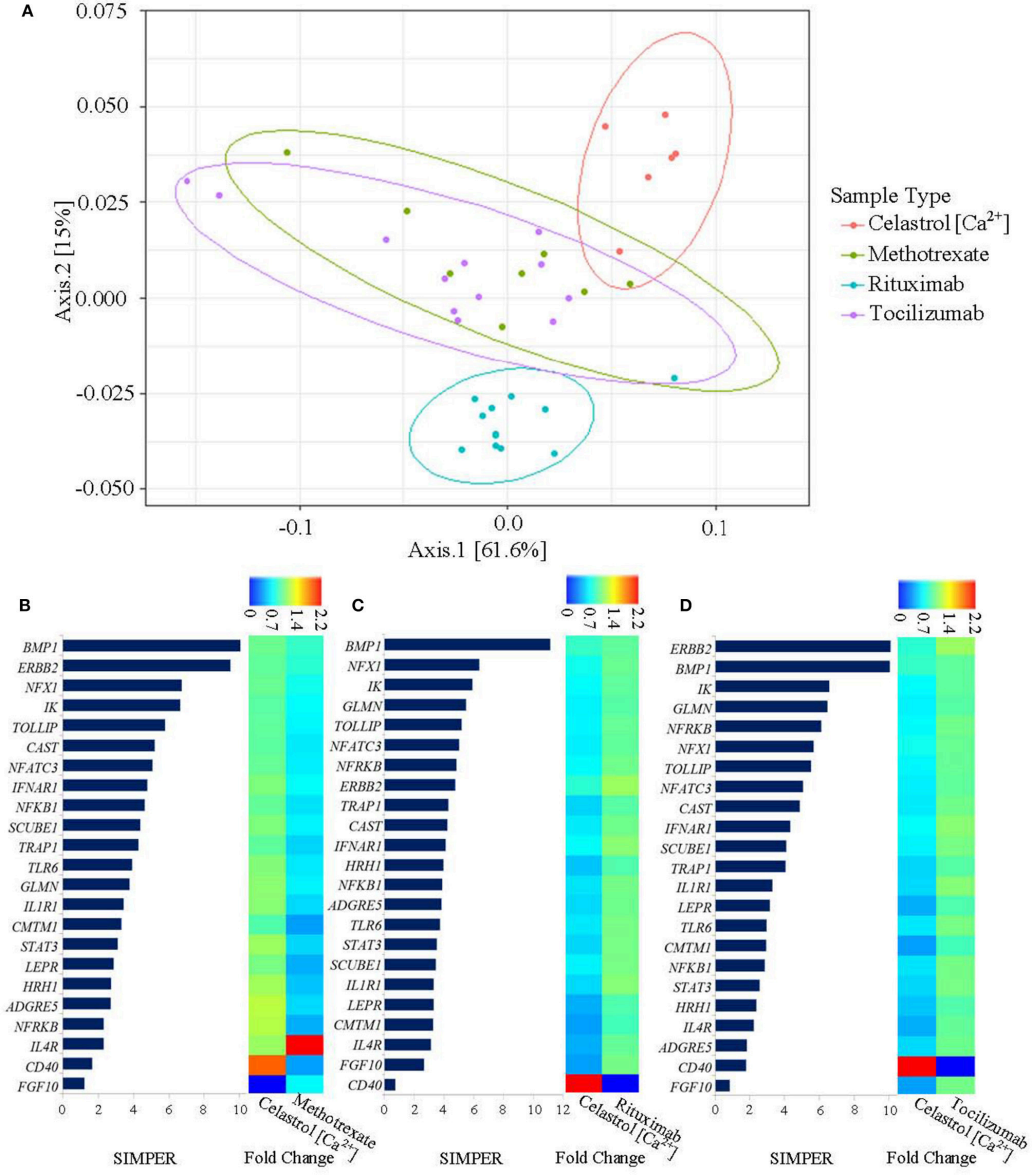
Comparative analysis of celastrol-mediated Ca^2+^ mobilization (celastrol-[Ca^2+^]) regulated genes with FDA-approved drug for RA. **(A)** Principal coordinates analysis with weighted UniFrac distance. Axes are showing percentile variations among the groups. Each dot corresponds to studied sample and respectively colored to sample types. **(B)** SIMPER analysis of the selected genes expression profile that are regulated by celastrol-[Ca^2+^] and methotrexate. **(C)** SIMPER analysis of the selected genes expression profile that are regulated by celastrol-[Ca^2+^] and rituximab. **(D)** SIMPER analysis of the selected genes expression profile that are regulated by celastrol-[Ca^2+^] and tocilizumab. X-axis is showing percentile similarity of the genes between the compared groups. The genes with higher percentage are more similar in responses toward celastrol-[Ca^2+^] and respective FDA-approved drug. The fold change of each gene after drug treatment has been shown in heat map.

In order to determine whether the mode of action of celastrol-[Ca^2+^] gene expression is novel mechanism for RA management, bioinformatics analysis was employed to compare the results obtained in this study with those from FDA-approved drugs. Three datasets were downloaded from NCBI database that were comprised of genes expression profile (before and after treatment) of RA patients with methotrexate, rituximab, and tocilizumab. The datasets were subset to the list of genes that matched with celastrol-[Ca^2+^] regulated genes and comparatively analyzed using UniFrac distance analysis. As shown in Figure 4A, celastrol-[Ca^2+^] differentially modulated the genes expression. However, celastrol-[Ca^2+^] regulated genes were comparable with changes observed after methotrexate and tocilizumab treatments, whereas, greater dissimilarity was observed between rituximab and celastrol-[Ca^2+^]. Moreover, the genes that responded similarly to celastrol-[Ca^2+^] and methotrexate (Figure 4B), celastrol-[Ca^2+^] and rituximab (Figure 4C), and celastrol-[Ca^2+^] and tocilizumab (Figure 4D) were determined using SIMPER analysis using Bray-Curtis method. These findings suggested that the celastrol-[Ca^2+^] associated anti-inflammatory mechanism could be a specific target for RA intervention.

## Discussion

In this study, we found that celastrol significantly downregulated the expression of a number of genes related to the control of RASFs cellular pathophysiology. In fact, the suppressive effects of celastrol in immunoregulatory genes expression have been documented. By using multiplex analysis, Venkatesha et al. demonstrated that celastrol can reduce the expression of cytokines, such as TNF-α and IL-1β, and chemokines including MCP-1, MIP-1α, and RANTES, in different immunocellular components collected from a murine adjuvant-induced arthritis (AIA) model (Venkatesha et al., 2012). However, the detail molecular linkages between celastrol and such inflammatory-associated signaling are yet to be defined. Our PCR array and quantitative PCR data validated that the expression of a significant number (close to 20%) of these inflammatory genes was manipulated by celastrol in a Ca^2+^-dependent manner. Most importantly, the expression of these genes, critical to the perpetuation of the inflammatory phenotype of RA, were sensitive toward cytosolic Ca^2+^ accumulation as illustrated by the BAPTA/AM treatment suggesting the efficacy of targeting cellular Ca^2+^ level in RA therapy. Intriguingly, CD40 receptor was the only upregulated gene upon Ca^2+^ mobilization amongst the reported genes. In fact, the engagement of CD40 ligand and RASFs-expressed CD40 receptor can activate RANKL induction via ERK-1/2, p38 MAPK, and NF-κB activation and result in osteoclast hyperplasia which leads eventually to cartilage and bone destruction (Lee et al., 2006). Such observation further suggested the significant role of cellular Ca^2+^ in RA pathogenesis, which also aroused the concern of adverse effects associated with celastrol, since CD40 upregulation may activate the downstream inflammatory pathways worsening the RA progression. However, as demonstrated in our network map, such inflammatory pathways could, at the same time, be downregulated by other celastrol-regulated genes. On the other hand, celastrol induced downregulation of LEPR, cognate receptor for the satiety factor leptin, implying the possibility of abnormal weight gain of animals or patients upon celastrol treatment. Recently, Liu et al., found that prolonged exposure of mice with celastrol lead eventually to weight loss of the animals without inducing any toxicity and that mice with desensitized leptin signaling phenotype presented no significant responses to celastrol in terms of their weight (Liu et al., 2015). Therefore, it is reasonable to conclude that administration of celastrol in a reasonable dosage will not create significant toxicity which suggested the therapeutic value of our findings. In fact celastrol treatment with the use of AIA rat model has been reported to mitigate inflammatory RA with no toxicity, including the liver and kidney damages, demonstrated (Cascão et al., 2015).

Many receptors and transporter proteins are responsible for maintaining cellular Ca^2+^ homeostasis (Schwaller, 2012), for example, sarco/endoplasmic reticulum Ca^2+^-ATPase (SERCA), inositol trisphosphate receptor (InsP_3_), and ryanodine receptor (RyR) which located on the ER are the key managers of intracellular Ca^2+^ storage. In addition, the sodium-calcium (Liu et al., 2015) exchanger NCX and uniporter on the mitochondria are another pair of intracellular Ca^2+^ receptors important for cytosolic Ca^2+^ regulation. On the plasma membrane, the plasma membrane Ca^2+^ ATPase (PMCA), together with, other Ca^2+^ channels such as voltage-operated calcium channel (VOCC), receptor-operated calcium channels (ROCC), and store-operated channels (SOCC), help to control the Ca^2+^ trafficking between cytoplasmic and extracellular environment. The observed Ca^2+^ flux in our RASFs is most probably due to the coupling of celastrol with SERCA (unpublished data) which further antagonizes the function of the receptor. The inactivation of SERCA stops the transportation of Ca^2+^ into the ER and results in cytosolic Ca^2+^ accumulation which mediates the expression of the validated genes. Upon celastrol treatment, *CAST, LEPR, TOLLIP*, and *TRAP1*, could be partially restored to the untreated level by BAPTA/AM implying that other unknown molecular mechanisms may underpin the regulatory machinery. After extensive literature review, the transcriptional regulations of *CD40, TRAP1, NFATC3*, and *CAST*, have been reported before which are related to cytosolic Ca^2+^ mobilization (Casanova et al., 2006; Landriscina et al., 2010), the remaining 19 genes appear to be newly discovered to have their expression machinery regulated by Ca^2+^ signaling.

The molecular pathways which are mediated by the products of our validated genes may form a complex network in RASFs with the constituting pathways crosstalk with each other. We accordingly generated a blueprint outlining such potential molecular circuitry using literature information documenting the signaling cascades that connect the products of individual validated gene to Ca^2+^ signaling, and RA pathogenesis. Although such signaling network is created by integrating the molecular pathways described in different cell types, they could potentially be involved in the regulatory machinery of RASFs which provided crucial hints for supporting follow-up functional experiments. Generally, these pathways could be summarized into five different categories of signaling cascade which are: (1) the calpains/calmodulin-calcineurin-NFATc3 axis; (2) PI3K-Akt/PKC-NF-κB axis; (3) JAK-Stat axis; (4) ERK-MMP axis; and (5) MyD88-AP1 axis. The calpains/calmodulin-calcineurin-NFATc3 axis is *per se* a well-known Ca^2+^-associated pathway inevitably regulating the cartilage catabolism and osteoclast formation, angiogenesis, and inflammation during RA pathogenesis (Sitara and Aliprantis, 2010). Upon Ca^2+^ influx, the calcium sensor proteins, including calpains and calmodulin, bind to the cation and activate calcineurin which further dephosphorylates NFATc3 (Im and Rao, 2004). After nuclear translocation, NFATc3 complexes with other cell-type specific transcription factors (TF) for regulating the downstream expression of genes, like RANKL, which is the molecular culprit causing bone erosion (Wu et al., 2007). Because of the capacity of NFATc3 to partner with other TF in various tissues or cellular components, the calpains/calmodulin-calcineurin-NFATc3-mediated gene expression could lead to a widespread of pathogenic effects. Our validated genes also include calpastatin which is the upstream inhibitor for calpains suggesting that cellular Ca^2+^ is holistically involving in the regulation of the various signaling pathways. Therefore, targeting the calpains/calmodulin-calcineurin-NFATc3 axis via the regulation of cytosolic Ca^2+^ distribution with Ca^2+^-mobilizing agent like celastrol, is a potential strategy for intervening RA progression. It is worth noting that, calpains could be the central hub in our molecular circuitry diagram intertwining the various signaling cascades. For example, calpains can intervene with the JAK-STAT axis by inhibiting the activity of suppressor of cytokine signaling 3 (SOCS3) (Pothlichet et al., 2008; Miyazaki et al., 2015). For the PI3K-Akt/PKC-NF-κB axis, calpains can regulate the pathway by inhibiting at the level upstream of NF-κB (Li et al., 2011, 2014; Storr et al., 2011, 2015). Also, calpains can manipulate the ERK-MMP axis by direct activation of ERK1/2 (Moshal et al., 2006). Although cellular and animal test are needed for further validation of our findings, other studies using AIA rat showed that celastrol can modulate the NF-κB pathway, MAPK pathway, and the JAK/STAT pathway (Venkatesha et al., 2016) which may support the signaling circuitry as proposed in this report. Also, the PI3K-Akt/PKC-NF-κB axis may significantly be regulated by celastrol-induced Ca^2+^ flux in RASF, since more than one third of the validated genes, including histamine receptor H1 (*HRH1*), *CD40, NFKB1, LEPR, IL4R, FGF10*, and *ERBB2*, potentially target this signaling axis with most of them functioned molecularly via PI3K, the upstream kinase of Akt and PKC. NF-κB involved extensively in the development of chronic inflammation (Tak and Firestein, 2001), our findings point toward the efficacy of controlling RA progression by regulating the balance of cellular Ca^2+^. On the other hand, the pathway mediating the toll-like receptor and IL-1α/βsignaling are also the potential target of the genes *TOLLIP, TLR6*, and *IL1R1* via the MyD88-AP1 axis (Dower et al., 1986; Burns et al., 1998, 2000; Bulut et al., 2001; Ahmad et al., 2007; de Almeida et al., 2013). The manipulation of such genes could, therefore, regulate the innate immune responses such as cellular migration and inflammation. There are several validated genes of which the corresponding signaling pathways cannot be well defined or lack of information acquired from the literature search. However, the products of these genes are known to participate in the development of inflammation like invasion, angiogenesis, cellular proliferation, and etc. The functions of these genes (*ADGRE5, BMP1, CMTM1, IK, NFX1*, and *SCUBE1*) and the associated molecular mechanisms underlying the corresponding signaling, therefore, deserve in-depth investigation for completing the signaling network as depicted in this study.

Taken together, we have discovered that cellular Ca^2+^ homeostasis is an important factor regulating the expression of a group of genes in RASFs which may significantly affect RA pathogenesis. As supported by the bioinformatics analysis, this Ca^2+^-dependent mechanism induced by celastrol is a new mode of action which has not been documented previously by using other conventional RA pharmaceutical interventions such as methotrexate, rituximab, and tocilizumab (Burmester and Pope, 2017; McInnes and Schett, 2017). However, the use of celastrol also demonstrated similarities to two out of three of the examined FDA-approved RA drugs (methotrexate and tocilizumab) in terms of the expression of some genes for example *BMP1* and *ERBB2* which support the therapeutic potency of celastrol in RA treatment. In addition, products of the celastrol induced Ca^2+^-mediated genes are participating in both upstream and downstream of molecular mechanisms leading to a comprehensive signaling control in a top-down basis not just targeting a single signaling messenger within a particular pathway. Therefore, our data highlighted the potential application of compounds, like celastrol, which are capable of regulating cytosolic Ca^2+^ level, as molecular tools for investigating the pathomechanisms of RA and other chronic inflammatory disorders. Our findings also provide forecasting platform for the establishment of functional experiments to validate our recent findings by using *in vitro* and *in vivo* models which facilitate the exploitation of novel pharmaceutical targets and therapeutic compounds.

## Author contributions

IdSRD and SM: conducted the experiments and drafted the manuscript. FG-M: prepared the figures and, materials and methods. IK and WH: conducted the bioinformatics analysis. BL: prepared the discussion and revised the whole manuscript. VW and LL: conceived the idea and designed the experimental plan.

### Conflict of interest statement

The authors declare that the research was conducted in the absence of any commercial or financial relationships that could be construed as a potential conflict of interest.

## Abbreviations

RA: Rheumatoid arthritis
RASF: rheumatoid arthritis synovial fibroblasts
PCR: polymerase chain reaction
ADGRE5: adhesion G protein-coupled receptor E5
BMP1: bone morphogenetic protein 1
CAST: calpastatin
CD40: cluster of differentiation 40
CMTM1: CKLF-like MARVEL transmembrane domain containing 1
ERBB2: Erb-B2 receptor tyrosine kinase 2
FGF10: fibroblast growth factor 10
GLMN: glomulin
HRH1: histamine receptor H1
IFNAR1: interferon α and β receptor subunit 1
IK: interferon inhibiting cytokine factor
IL1R1: interleukin 1 receptor type 1
IL4R: interleukin 4 receptor
LEPR: leptin receptor
NFATC3: nuclear factor of activated T-cells 3
NFKB1: nuclear factor κB subunit 1
NFRKB: nuclear factor related to κB binding protein
NFX1: nuclear transcription factor, X-box binding 1
SCUBE1: signal peptide, CUB domain, EGF-like domain-containing protein 1
STAT3: signal transducer and activator of transcription 3
TLR6: toll-like receptor 6
TOLLIP: toll interacting protein
TRAP1: TNF receptor-associated protein 1.
