# Supplementary material for: The Calcium-Induced Regulation in the Molecular and Transcriptional Circuitry of Human Inflammatory Response and Autoimmunity
Source: Front Pharmacol. 2018 Jan 8;8:962. doi: 10.3389/fphar.2017.00962 (PMC5766673; doi:10.3389/fphar.2017.00962)
Supplement: Supplementary file 1 [file DataSheet1.DOCX]

Supplementary Material

**The calcium-induced regulation in the transcriptional and molecular circuitry of human inflammatory response and autoimmunity**

**Ivo Ricardo de Seabra Rodrigues Dias^1#^, Simon Wing Fai Mok^1#^, Flora Gordillo Martínez^1^, Imran Khan^1^, Wendy Wen Luen Hsiao^1^, Betty Yuen Kwan Law^1^, Vincent Kam Wai Wong^1*^and Liang Liu^1*^**

^1^ State Key Laboratory of Quality Research in Chinese Medicine, Macau University of Science and Technology, Macau, China.

**^#^** Ivo Dias and Simon Mok contributed equally to this work.

*** Corresponding authors:** Prof. Liang Liu and Dr. Vincent Kam Wai Wong

Address correspondence: State Key Laboratory of Quality Research in Chinese Medicine, Macau University of Science and Technology, Avenida Wai Long, Taipa, Macau, China

Tel.: +853-8897-2238 (LL); +853-8897-2408 (VKWW)

Fax:+853-2882-3312 (LL); +853-2882-2799 (VKWW)

E-mail address: lliu@must.edu.mo (LL); bowaiwong@gmail.com (VKWW)

# Supplementary Data

**Supplementary Video.** Single cell imaging monitorization of intracellular cytosolic Ca^2+^ dynamic in celastrol-stimulated RASFs. Cells treated with 1 µM celastrol were loaded with FLIPR Calcium 6 dye. Representative view of intracellular Ca^2+^ mobilization signal utilizing Applied Precision DeltaVision Elite in real-time mode. The mean intensity of fluorescence signal at 525 nm is shown in **Figure 1B**.

# Supplementary Table

## Supplementary Table. Primer sequences designed for the primers utilized for validation of genes.

| **Housekeeping Gene** | **Primer sequence** | |
| --- | --- | --- |
| **GAPDH** | Forward | 5' - CAGCCTCAAGATCATCAGCA - 3' |
|  | Reverse | 5' - TGTGGTCATGAGTCCTTCCA - 3' |
| **Gene of interest** | **Primer sequence** | |
| **ADGRE5** | Forward | 5' - CTTTCCCGATTCTTCGACAA - 3' |
|  | Reverse | 5' - TTCCATCAGTTCATCCACCA - 3' |
| **BMP1** | Forward | 5' - CAACACGTTCGGCAGTTATG - 3' |
|  | Reverse | 5' - CACTGGTGGATGTCACCTTG - 3' |
| **CAST** | Forward | 5' - GAAGTCGATGAGGCAAAAGC - 3' |
|  | Reverse | 5' - CATCTTTATCCGTGGCTGGT - 3' |
| **CD40** | Forward | 5' - GAGATCAATTTTCCCGACGA - 3' |
|  | Reverse | 5' - CGACTCTCTTTGCCATCCTC - 3' |
| **CMTM1** | Forward | 5' - ATCACCCAAGCCAATGAGTC - 3' |
|  | Reverse | 5' - GCAAGTGGTGAAGGGTTAGC - 3' |
| **ERBB2** | Forward | 5' - AACCAGCTGGCTCTCACACT - 3' |
|  | Reverse | 5' - CAGAACTCTCTCCCCAGCAG - 3' |
| **FGF10** | Forward | 5' - CCATGAACAAGAAGGGGAAA - 3' |
|  | Reverse | 5' - TGCTGCCAGTTAAATGATGC - 3' |
| **GLMN** | Forward | 5' - GGGCACACAGACCAGCTATT - 3' |
|  | Reverse | 5' - AAGGCATCGAACAACAGGAC - 3' |
| **HRH1** | Forward | 5' - ACCCCCAAGGAGATGAAATC - 3' |
|  | Reverse | 5' - GCCTGCATGTGCACAATATC - 3' |
| **IFNAR1** | Forward | 5' - AAGCTCAGATTGGTCCTCCA - 3' |
|  | Reverse | 5' - CCATCCAAAGCCCACATAAC - 3' |
| **IK** | Forward | 5' - CAAAGGACCTGGGTCTACCA - 3' |
|  | Reverse | 5' - TTCTGGCTTCTTCAGCGATT - 3' |
| **IL1R1** | Forward | 5' - ATTGATGTTCGTCCCTGTCC - 3' |
|  | Reverse | 5' - TGAATCCTGGAGGCTTGTTC - 3' |
| **IL4R** | Forward | 5' - CCGCCTCGTGGCTATAATAA - 3' |
|  | Reverse | 5' - CAGGGCAAGAGCTTGGTAAG - 3' |
| **LEPR** | Forward | 5' - AATGCATTTTCCAGCCAATC - 3' |
|  | Reverse | 5' - TGGCTTCACCACAGAATCAG - 3' |
| **NFATC3** | Forward | 5' - TGTTATGCTGGGTCCCTTTC - 3' |
|  | Reverse | 5' - CAGAAGCATTGAGCCACGTA - 3' |
| **NFKB1** | Forward | 5' - GCCTCTAGATATGGCCACCA - 3' |
|  | Reverse | 5' - TCAGCCAGCTGTTTCATGTC - 3' |
| **NFRKB** | Forward | 5' - TCGAGCTGTTCCTTCCAGTT - 3' |
|  | Reverse | 5' - GGAAGAGGGCAGCTAATTCC - 3' |
| **NFX1** | Forward | 5' - CCAAGTTTATGGCCTGGAGA - 3' |
|  | Reverse | 5' - TTTCAAGCACACCTGTCAGC - 3' |
| **SCUBE1** | Forward | 5' - CTCATCGTGGTCCCTGAGAT - 3' |
|  | Reverse | 5' - CATAGGTGGTGATGGACGTG - 3' |
| **STAT3** | Forward | 5' - GGCCATCTTGAGCACTAAGC - 3' |
|  | Reverse | 5' - CTGGGTCTTACCGCTGATGT - 3' |
| **TLR6** | Forward | 5' - ACTGACCTTCCTGGATGTGG - 3' |
|  | Reverse | 5' - CTGGCAGCTCTGGAAGAAAT - 3' |
| **TOLLIP** | Forward | 5' - CTGAAAGCCATCCAGGACAT - 3' |
|  | Reverse | 5' - ATCTGCAGCAGGGAGTTGAT - 3' |
| **TRAP1** | Forward | 5' - CTTGGAAAAACTGCGTCACA - 3' |
|  | Reverse | 5' - GATGGTGATGGTGCCTTTCT - 3' |
